# Ventricular septal defect in a child with Alport syndrome: a case report

**DOI:** 10.1186/1471-2261-10-48

**Published:** 2010-10-05

**Authors:** Pier Paolo Bassareo, Andrea Raffaele Marras, Giuseppe Mercuro

**Affiliations:** 1 Department of Cardiovascular and Neurological Sciences, University of Cagliari, Cagliari, Italy; 2 Study Center for Cardiac Disease in Pediatric Age, University of Cagliari, Cagliari, Italy

## Abstract

**Background:**

Alport syndrome (AS) is a rare inherited disorder characterized by an inflammation of the kidneys and damage to the glomerular capillaries, ultimately leading to renal failure at an early age. To date, rare reports of cardiac involvement in AS have been described, due in the majority of cases to the higher risk of heart conduction abnormalities in these patients, at times requiring implantation of a transcutaneous pacemaker. An increased risk of hypertension is likewise commonly featured.

**Case presentation:**

We report the case of a 17-year-old female affected by a very severe early form of AS. A previously unreported association of the syndrome with congenital heart disease (CHD), (in this case membranous ventricular septal defect), is also reported. A possible pathophysiological mechanism underlying the concomitant manifestation of these two disorders is suggested. Complications implicated in surgical treatment of CHD are described. Clinical and therapeutic management of AS with cardiovascular involvement are discussed, and a short literature review performed.

**Conclusions:**

This first report of a cardiovascular association highlights the possible involvement of collagen mutations in the two pathologies. Even when drug-resistance appears to be responsible for the failure to control secondary hypertension in AS, clonidine may represent a safe, effective option in the normalization of high blood pressure.

## Background

Alport syndrome (AS), first described by Cecil A. Alport in 1927, is a rare inherited form of progressive kidney inflammation (0.3 - 2.3% of patients presenting with end-stage renal disease) affecting predominantly males. The disorder is caused by defects in the genes encoding a connective tissue protein, one of the several subunits of collagen (particularly type IV), and a major component of the renal glomerular basal membrane [1-5]. Various mutations lead to a broad spectrum of significantly different disease phenotypes, ranging from benign mild renal insufficiency to end-stage renal disease. AS is inherited by the majority of patients (approx 85%) as an X-linked trait, although some patients display an autosomal recessive (10-15%) or autosomal dominant (rare) genetic transmission [6]. As a general rule, severity of the disease is greater in males with X-linked AS, being equally severe in male and female homozygotes featuring the autosomal recessive form [7,8]. AS should be differentiated from other types of chronic glomerulopathies [9]. To this regard, microscopic renal changes in AS are frequently observed in early biopsy specimens of renal tissue featuring podocyte hypertrophy and stiffness of the capillary wall, associated or not with the presence of tubular red blood cell casts. Focal and segmental thickening of the glomerular basal membrane may also at times present with progressive enlargement of mesangial stalks. Development of segmental, and subsequently diffuse, glomerular sclerosis in an increasing number of glomeruli leads to complete sclerosis. An increase in size and severity of foci of tubulo-interstitial lesions may precede marked glomerular changes. Clusters of interstitial foam cells frequently found in proteinuric patients tend to decrease in end-stage renal disease. No significant arterial changes are initially observed [3]. Hematuria, the most common and earliest manifestation of AS, usually occurs in the first years of life, while proteinuria is absent and develops throughout childhood into the above described more aggressive forms. The disease generally progresses with age [10]. Hypertension is commonly observed in patients affected by AS, with both incidence and severity increasing with age and degree of renal failure [11]. Due to collagen involvement in ear and eye development, hearing and ocular abnormalities are also present. Deafness is never present at birth, but may be manifested in late childhood or early adolescence, although some patients affected by AS feature severe nephropathy but normal hearing. Ocular abnormalities include anterior lenticonus, dot-and-fleck retinopathy (85%), and posterior polymorphous corneal dystrophy. Other systemic manifestations (in particular leiomyomatosis, and blood disorders) have also been described [6,12]. No established form of treatment currently exists for AS. Some patients benefit from angiotensin-converting enzyme inhibitors or angiotensin receptors blockers resulting in the reduction of proteinuria and progression of renal disease [13-15]. A strict control of blood pressure is paramount. Several literature reports describe the ability of cyclosporine in reducing proteinuria and stabilizing renal functions, although diverging opinions are reported and further studies should be undertaken to better define the effect of the drug in AS patients [16,17]. In patients with severe renal failure, dialysis treatment is usually recommended. Moreover, subjects with end-stage renal disease invariably undergo kidney transplant, with 3-5% of patients developing an autoimmune reaction in the first year after transplant (anti-glomerular basal membrane nephritis) with loss of allograft [18]. Here we report the case of a female adolescent with a previously undescribed association of AS with congenital heart disease (CHD), i.e. a membranous ventricular septal defect (VSD). The possible pathophysiological mechanism underlying these two disorders is hypothesized. Surgical treatment of VSD and the complications arising is described, and the clinical and therapeutic management of AS discussed.

## Case presentation

**Figure 1:**
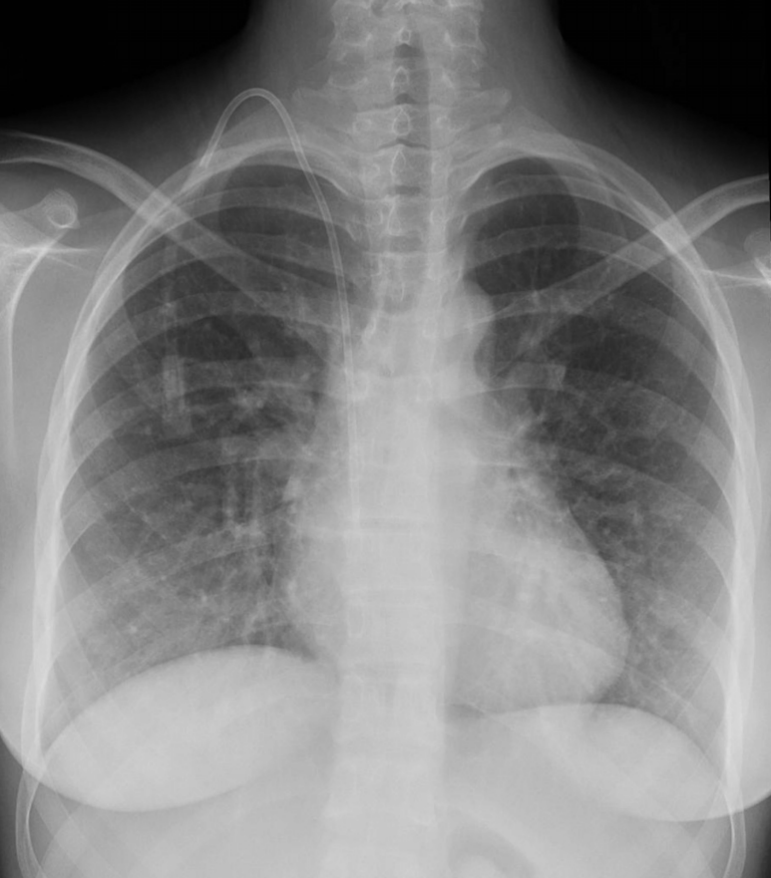
**Chest antero-posterior X-ray showing a normal heart size, and a more marked pulmonary vasculature, particularly at basal level**. A central venous catheter is evident as well.

**Figure 2:**
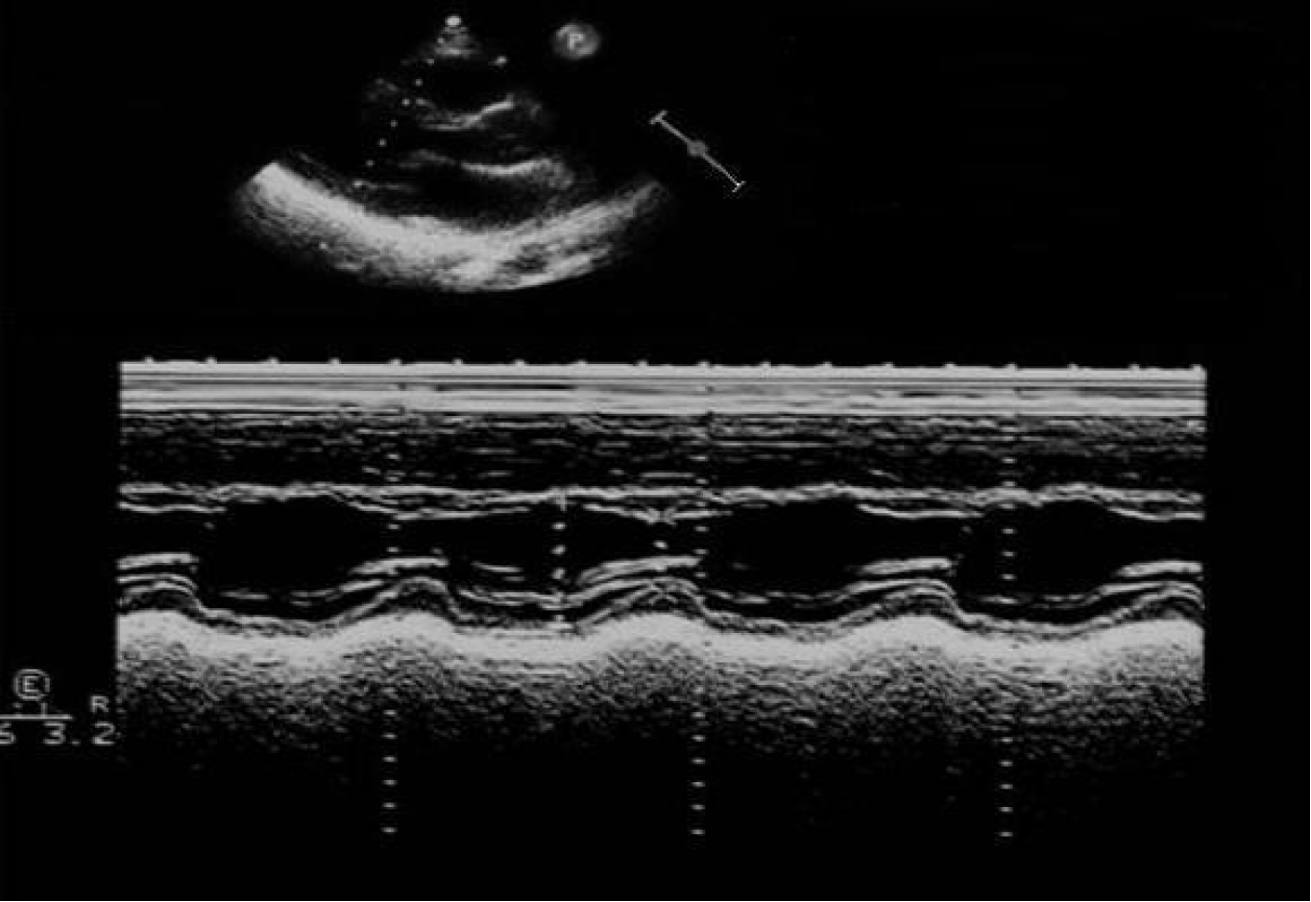
**Transthoracic echocardiogram: parasternal log axis view showing septal paradoxical movement at M-mode modality**.

**Figure 3:**
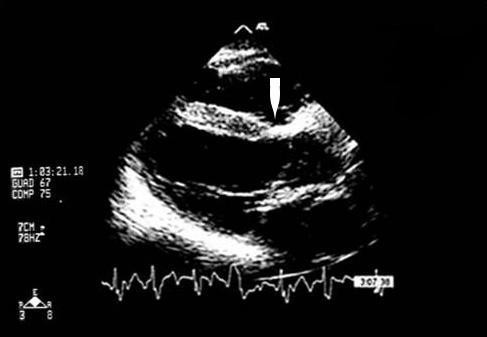
**Closure of the ventricular septal defect by a patch (white arrow)**.

**Figure 4:**
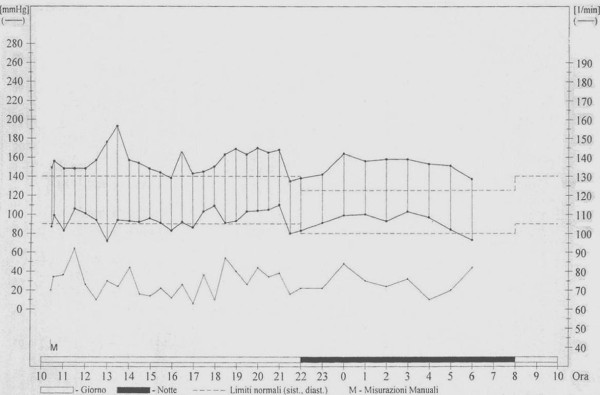
**24-hour ambulatory blood pressure monitoring profile showing a persistent elevation of both systolic and diastolic blood pressure**. The physiological decline in nocturnal pressure was absent (non-dipper pattern)

A 17-year-old female was referred to our clinical Centre due to difficulty in controlling her blood pressure by means of treatment with angiotensin-converting enzyme (ramipril 10 mg/day), angiotensin receptor blockade (losartan 100 mg/day), calcium antagonist (nifedipine 10 mg/day) and alpha lithic (doxazosin 4 mg/day). The patient was affected by an early onset, extremely aggressive form of AS (X-linked type, due to a nonsense mutation in the COL4A5 gene), with development of severe renal insufficiency requiring treatment with dialysis (three sessions/week). Additionally, the patient had a history of moderate membranous VSD treated by surgery at the age of one year. Physical examination underlined the minuteness of the subject (height 151 cm; weight 44 Kg; BMI 19.3 Kg/m^2^). Systemic blood pressure was 157/94. The patient presented with perimalleolar oedema of the legs. Heart sounds were rhythmic with no murmur. Basal lung auscultation revealed crackling wheezes. Electrocardiogram revealed sinus rhythm with heart rate 92 beats/min. Chest X-ray revealed a normal heart size, and a more marked pulmonary vasculature, particularly at basal level (Figure 1). Transthoracic echocardiography revealed complete surgical closure of VSD, with no signs of intracardiac shunt (Figures 2, and 3). The two ventricular chambers were well balanced and pulmonary arterial pressure - calculated from tricuspid valve insufficiency - was normal. Surgery performed to close the VSD with a bovine pericardial patch had been complicated by development of a transient complete atrioventricular block lasting seven days and promptly treated with a temporary pace maker. The patient was discharged from hospital two weeks after surgery in good clinical conditions. A 24-hour ambulatory blood pressure monitoring (ABPM) was performed to better define daily blood pressure variability, in particular to avoid a possible white coat effect, and evaluate drug-resistance. ABPM analysis (Figure 4) highlighted a persistent elevation in both systolic and diastolic blood pressure. Physiological decline in nocturnal pressure was also absent (non-dipper pattern). As treatment prescribed was not capable of adequately controlling the patient's blood pressure, add-on therapy was introduced (2.5 mg/week transdermal clonidine patch applied to the skin every 7 days). After two weeks the patient's blood pressure had normalized. Following the development of cutaneous rush at the site of patch application, after 1 month transdermal clonidine was replaced with oral clonidine (150 mg × 3/day), achieving the same satisfactory effect in controlling blood pressure.

## Discussion

Cardiac involvement in AS is very rare. Literature reports refer a higher risk of heart conduction abnormalities in patients with AS. Even patients with a normal preoperative electrocardiogram or no conduction system disorders may present some degree of atrioventricular block, including complete atrioventricular block [19]. Generally speaking, this could reflect [19]:

a. an increase in conduction disorders caused by renal failure due to abnormalities in calcium metabolism leading to fibrosis and myocardial calcification;

b. high serum potassium levels. Although sinoatrial and atrioventricular nodes are apparently less sensitive to hyperkalemia than other cardiac fibers - owing to their calcium-dependent electrophysiological properties - atrioventricular block due to hyperkalemia may occur;

c. an external sympathetic block due to anaesthesia.

Furthermore, surgery for VSD closure is frequently complicated by the development of atrioventricular blocks. However, AS patients may have an additional risk in developing this surgical complication. In such cases, including the case described here, a transcutaneous pacemaker provides rapid and effective treatment in the operating room, thus facilitating the scheduling of definitive treatment. In addition, isolated case reports of arterial disease in males with AS, including dissection and aneurysm have been published. Immunohistochemistry bioptic findings have confirmed the abnormal absence of several type IV collagen chains from the aortic media of these patients. Accordingly, the routine screening of males with AS for aortic abnormalities may be clinically indicated [20]. To date however, and to the best of our knowledge no association between AS and any forms of CHD has been described. In our patient, the presence of a moderate membranous VSD had produced a left to right shunt, initially treated by diuretic therapy (furosemide 1 mg/Kg × 2/day). At the age of one year, surgical treatment was recommended and the VSD closed by means of a pericardial patch. As stated above, surgery was complicated by the development of a transient atrioventricular block, treated with a temporary pace maker. With regard to the possible pathophysiological mechanism underlying the association of AS and VSD, it should be borne in mind that the membranous portion of the interventricular septum consists of tough collagenous fibrous connective tissue, thus suggesting the involvement of collagen formation defects in the pathogenesis of membranous VSD [21,22]. Even if type IV collagen is mainly involved in glomerulosclerosis of AS kidneys, it would seem the mutations affecting the Alport gene also have secondary effects on the distribution of other types of collagen at the level of glomerular basal membrane constituents. These other types, which may also be involved in the development of VSD, might just be the link between these two so apparently different pathologies.

However, the above mechanism is merely putative and further studies should be undertaken to better clarify the relationship between the two disorders. This will likely not be an easy task due to the rarity of Alport disease. With regard to cardiovascular involvement in AS, high blood pressure is a common feature manifested secondary to renal failure[23]. In our patient, blood pressure was only normalized following the addition of clonidine to the previously established pharmacological therapy. Clonidine, which is not a first line drug in most cases of hypertension, is a direct-acting α2 adrenergic agonist that controls high blood pressure through stimulation of α_2 _receptors in the brain, leading to a decrease in cardiac output and peripheral vascular resistance, in turn resulting in a lowering of blood pressure. The drug may however produce side effects such as lightheadedness, dry mouth, dizziness, constipation, and hypotension. Clonidine also features peripheral alpha agonist effects, which may lead to the onset of hypertension. The latter effects may be observed following an overdose in children, as blood pressure increases. As clonidine is eliminated by the body, its peripheral effects wear off, and central hypotensive effects become visible. Both hypertensive and hypotensive effects may be harmful. Indeed, sudden discontinuation of the drug may elicit rebound hypertension due to a rebound in sympathetic outflow [24]. To this regard the utility of ABPM in monitoring daily blood pressure variability even in patients with end-stage renal disease undergoing dialysis is evident [25].

Lastly, the present case report is of particular interest in view of the high degree of severity of AS in such a young female, as the more severe forms of X-linked AS are generally manifested in males, with a later onset of end-stage renal disease.

## Conclusions

In conclusion, this previously unreported association of AS with CHD has contributed towards furthering insight into this rare disease. A mutation at the level of collagen synthesis may explain the concomitant presentation of the two pathologies. Even when drug-resistance appears to underlie the failure to control secondary hypertension in AS, clonidine may represent a safe, effective addition capable of normalizing high blood pressure [26].

## Abbreviations

AS: Alport syndrome; CHD: congenital heart disease; VSD: ventricular septal defect; ABPM: 24-hours ambulatory blood pressure monitoring

## Competing interests

The authors declare that they have no competing interests

## Authors' contributions

PPB: acquisition of data, conception and design - ARM: revising the manuscript critically - GM: final approval of the version to be published. All authors have read and approved the final manuscript.

## Pre-publication history

The pre-publication history for this paper can be accessed here:

http://www.biomedcentral.com/1471-2261/10/48/prepub
